# An Open-Source and Low-Cost Monitoring System for Precision Enology

**DOI:** 10.3390/s141223388

**Published:** 2014-12-05

**Authors:** Salvatore Filippo Di Gennaro, Alessandro Matese, Mirko Mancin, Jacopo Primicerio, Alberto Palliotti

**Affiliations:** 1 Istituto di Biometeorologia (IBIMET-CNR), Consiglio Nazionale delle Ricerche, Via G. Caproni 8, Firenze 50145, Italy; E-Mails: a.matese@ibimet.cnr.it (A.M.); j.primicerio@ibimet.cnr.it (J.P.); 2 Dipartimento di Scienze Agrarie, Alimentari e Ambientali, Università degli Studi di Perugia (UNIPG), Borgo XX Giugno 74, Perugia 06128, Italy; E-Mail: alberto.palliotti@unipg.it; 3 Dipartimento di Ingegneria dell'Informazione, Università di Parma (UNIPR), Viale G.P. Usberti, 181/A, Parma 43124, Italy; E-Mail: mirko.mancin@studenti.unipr.it; 4 Dipartimento Scienze Agrarie, Forestali e Alimentari, Università degli Studi di Torino (UNITO), Via Leonardo da Vinci 44, Torino 10095, Italy

**Keywords:** precision enology, wireless sensor network, open-source, Arduino, wine

## Abstract

Winemaking is a dynamic process, where microbiological and chemical effects may strongly differentiate products from the same vineyard and even between wine vats. This high variability means an increase in work in terms of control and process management. The winemaking process therefore requires a site-specific approach in order to optimize cellar practices and quality management, suggesting a new concept of winemaking, identified as Precision Enology. The Institute of Biometeorology of the Italian National Research Council has developed a wireless monitoring system, consisting of a series of nodes integrated in barrel bungs with sensors for the measurement of wine physical and chemical parameters in the barrel. This paper describes an open-source evolution of the preliminary prototype, using Arduino-based technology. Results have shown good performance in terms of data transmission and accuracy, minimal size and power consumption. The system has been designed to create a low-cost product, which allows a remote and real-time control of wine evolution in each barrel, minimizing costs and time for sampling and laboratory analysis. The possibility of integrating any kind of sensors makes the system a flexible tool that can satisfy various monitoring needs.

## 1. Introduction

Wine composition and quality are due to many different intrinsic and extrinsic variables, and high-quality grape production does not necessarily translate directly into high-quality wine. During the winemaking process, a complex series of chemical-physical and microbiological processes transform grapes and must into wine. Wine characteristics can frequently differ strongly between products from the same vineyard and even between wine vats, due to small differences in the development of those processes. This high variability means an increasing demand for control and process management and, as a result, higher costs. As in Precision Viticulture, the winemaking process requires a site-specific methodology, in order to optimize cellar practices and quality management. This kind of approach suggests a new concept of winemaking, which we therefore refer to as Precision Enology. Every stage of the production chain requires a careful process control, but manual sampling and measurements are time-consuming and frequently error-prone. For example, temperature is a critical variable during the early stage of the winemaking process, due to the fact that alcoholic fermentation is an exothermic reaction that must be constantly monitored in order to avoid serious problems. While large wineries frequently utilize steel fermenting vats equipped with temperature sensors, the smaller ones generally perform temperature measurements manually to reduce the costs of deploying wired sensors and actuation infrastructure. Many authors have suggested low-cost solutions to this using wireless temperature monitoring systems [1–4]. However, a correct winemaking monitoring needs to control many other processes and consequently many other parameters in addition to temperature. After the fermentation phase, which generally takes place in steel vats, wine undergoes an aging period in wooden barrels. Unlike the huge steel fermentation vats, small barrels (225 L) cannot be equipped with sensors and a power supply, due to the large number of units present in the wineries, and the need for periodic movement. Barrels are often stacked at least five or six high, obliging operators to work under hazardous conditions in order to sample and monitor the wine. For this reason, Di Gennaro *et al.* [5] suggested a novel solution for a wireless monitoring system to control malolactic fermentation (MLF) in barrel-stored wine. MLF is a metabolic reaction that commonly occurs after the alcoholic fermentation, and its principal effect is an improvement of the taste and flavor characteristics of the wine [6], but it can also promote the production of undesirable and toxic compounds [7]. The first prototype proposed in 2013 was a device housed in the silicone bung of the barrel, equipped with a temperature and pH sensor, that collected and sent data to a remote base unit.

This paper describes an open-source evolution of that prototype, in order to obtain a new instrument at a lower cost and with increased versatility. The system, named WineDuino, could support a large number of sensors, representing an easy tool for monitoring all winemaking processes.

## 2. Experimental Section

### 2.1. System Design

**Figure 1.**
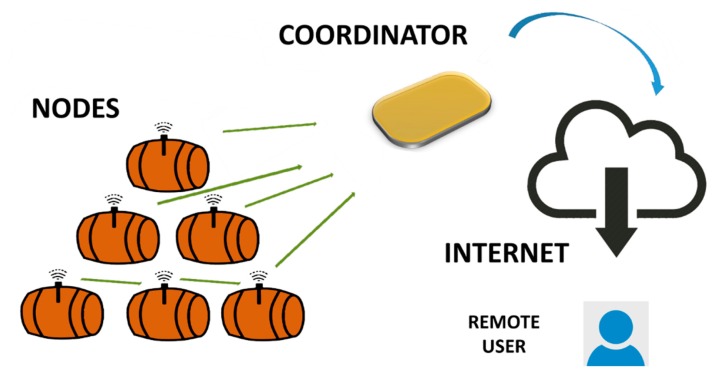
WineDuino system data transmission.

The WineDuino system consists of a series of nodes integrated in the silicone bungs, allowing direct monitoring of enological parameters during wine aging in barrels. Nodes acquire data and provide a wireless transmission to a coordinator unit, which collects and forwards data to a remote server (Figure 1).

### 2.2. Hardware Technical Details

#### 2.2.1. WineDuino Node

**Figure 2.**
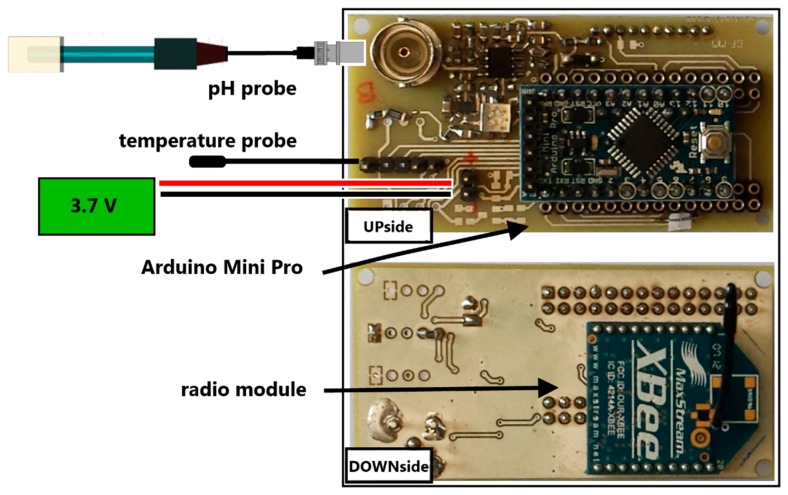
Scheme of WineDuino node hardware.

The nodes have been developed and built with Arduino open-source technology. The core is an Arduino Mini Pro board [8], designed and manufactured by SparkFun Electronics (Boulder, CO, USA), which is based on the ATmega328 microcontroller (Atmel Corporation, San Jose, CA, USA). It is of minimal size (approximately 18 mm × 33 mm) and provides 14 digital and eight analog channels with 10-bit resolution, which allow a large number of sensors to be managed. There are two versions of the Mini Pro, one runs at 3.3 V and 8 MHz, the other at 5 V and 16 MHz; for the WineDuino system the first one was chosen to optimize power management. The board comes without pre-mounted headers, allowing the use of various types of connectors or direct soldering of wires, so the microcontroller was programmed through a six-pin header connected to an FTDI cable, to provide USB power and communication with the board. The board node (Figure 2) acquires data from the sensors at four hourly frequency, and after readings, data are sent to the coordinator unit through the Xbee S1 radio module, which ensures a 30 m indoor range and provides a fast 250 Kbps RF data rate. This small module (27 mm × 24 mm) integrates a small wire antenna with 1mW transmit power. It requires 2.8–3.4 V power supply and typically consumes 50 mA or 10 μA in sleep mode. Xbee Series 1 offers six 10-bit ADC input and eight digital I/0, with AT and APi commands local or over-the-air configuration and serial data interface CMOS UART. It comes standard with 802.15.4 firmware at 2.4 GHz and easily provides point-to-point, star and mesh (with DigiMesh firmware) network topologies.

**Figure 3:**
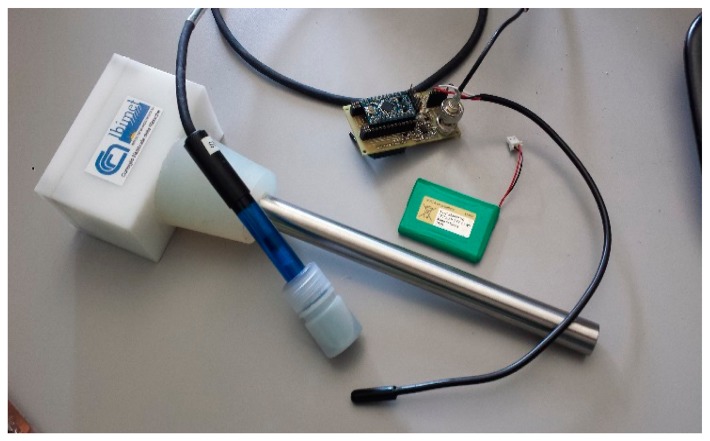
WineDuino node with sensor and dipstick.

Each node is powered by a 3.7 V at 2300 mAh Li-Po battery. A shield board was developed ad hoc to support Arduino Mini Pro and Xbee S1 radio modules, and provide slots for power supply and sensors. The first application of WineDuino system was to measure temperature and pH for monitoring MLF in wine. To provide compatibility with different commercial pH probes, an amplifier circuit with BNC connector and a trimmer were mounted on the board allowing the readings to be calibrated according to the construction parameters. Node casing is an IP65 plastic box (100 mm × 50 mm × 50 mm) mounted over the silicone bung. Sensors are housed in a stainless steel dipstick (AISI 304), which passes through the bung with the sensors at the end in direct contact with the wine, approximately in the middle of the barrel (Figure 3).

The dipstick (300 mm length × 20 mm diameter) is physiologically inert and suitable for food contact. For the temperature probe, the DS18B20 (Dallas Semiconductor Corp., Dallas, TX, USA) was chosen, a 1-wire digital sensor, characterized by low cost and short time response. It provides 9 to 12 bit temperature measurements, in an operating range of −55 °C to 125 °C, with ±0.5 °C precision. The pH measurement is taken by a 3550-ASP200-2-1M-BNC pH Lab Electrode (Phidgets Inc., Calgary, AB, Canada), it is an economical combination electrode ideal for general-purpose applications, with impact-resistant epoxy body. The electrode provides a fast, stable response and is suitable for prolonged pH readings. The sealed, gel-filled design requires virtually no maintenance, which is a prerogative for this kind of application. The probe measures 120 mm (length) × 12 mm (diameter), can withstand temperatures up to 80 °C measuring pH levels from 0 to 14, and it provides a BNC connector. WineDuino system sensors were calibrated following the protocol proposed in Di Gennaro *et al.* [5].

#### 2.2.2 Coordinator Unit

**Figure 4.**
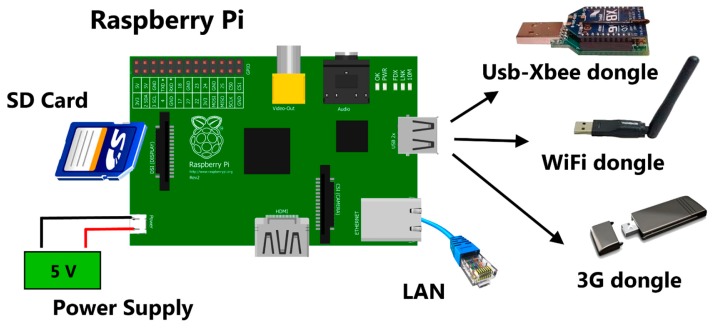
Scheme of coordinator unit hardware.

The coordinator unit (Figure 4) is realized with a Raspberry Pi board model B [9], a small-sized single-board computer (86 mm × 54 mm), which is an open-source project developed in the UK by the Raspberry Pi Foundation (Mitchell Wood House, Caldecote, UK), intended to run a Linux kernel based operating system. The design is based on a Broadcom BCM2835 SoC (Broadcom Corporation, Cambridge, UK), which includes an ARM1176JZF-S 700 MHz processor, VideoCore IV GPU, 512 Mb of RAM, and an SD card for booting and storage media for the data received from nodes. Moreover, the board provides one 10/100 Ethernet and two USB ports. It requires 5 V power supply via microUSB or GPIO header, and is located on a sidewall of the cellar inside an IP65 plastic box to ensure humidity and dust protection. The coordinator unit is equipped with a USB-Xbee dongle (Xbee S1 radio module), which allows an 802.15.4 network communication with the node Xbee modules. Data collected are transmitted to the web server using the second USB port, through a 3G key or a WiFi dongle that will connect to an existing wireless connection, but eventually also via Ethernet.

### 2.3. Communication Protocol

The system provides two types of communication protocols. For data transmission from node unit to coordinator unit, the system employs Xbee modules Series 1 that use the IEEE 802.15.4 networking protocol for fast point-to-multipoint or peer-to-peer networking. The time it takes to transmit a data packet is a sum of the Time on Air and Time for Carrier Sense Multiple Access—Collision Avoidance (CSMA-CA) and Retries. The time on the air taken to send payload bytes depends on the RF band rate of 250 Kbps, which is a 4 μs bit time or 32 μs byte time. CSMA-CA means that before a radio begins transmitting on the air it senses the carrier channel to make sure the air waves are clear (called CCA-Clear Channel Assessment). If it senses strong enough activity on the channel, it will perform a random delay and then try again with another CCA. Once data are received from the coordinator unit, they are transmitted to the remote server database with Wi-Fi technology based on IEEE 802.11 or 3G mobile telecommunications technology protocols. Data are stored in a MySQL database, which can be queried by many clients to provide textual data tables, graphical visualization plots and export data function in .csv format. The user can view data remotely via a browser or a dedicated app.

### 2.4. Software

The software was developed with different languages for each part of the system. The nodes were programmed with Arduino IDE (Integrated Development Environment) [10], an open-source and extensible software, which allows easy code implementation to be written for data acquisition and transmission management. The coordinator unit is a server-like application software written in PHP, and thanks to ad-hoc developed API, it is possible to control data flow, in terms of collection from nodes Xbee modules, raw data elaboration and transmission to the database. When data are received from the coordinator unit, they are formatted in a JSON string, which will then be saved in the database to allow a dynamic data management as table or graphic visualization. The output string contains information about date, time, node ID number, pH and temperature values (unit and °C), and battery level (V).

## 3. Results and Discussion

**Figure 5.**
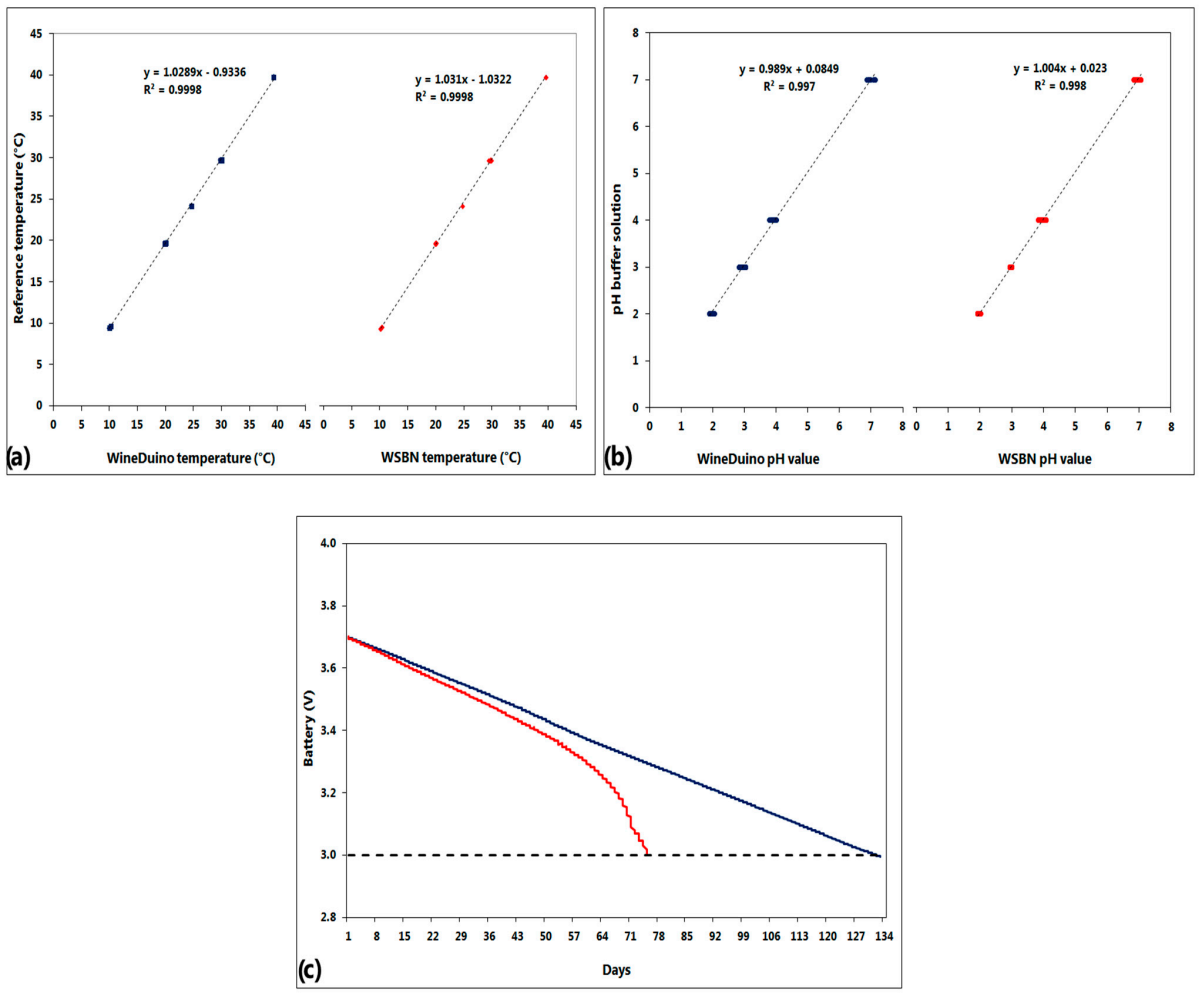
Performance comparison analysis between WineDuino system (blue) and WSBN (red) presented in the previous paper. (**a**) Temperature sensor calibration; (**b**) pH sensor calibration and (**c**) power supply test.

The system was analyzed in the laboratory in order to evaluate performance in terms of power consumption and sensors accuracy compared to the previous prototype. A cost analysis was also conducted to estimate the economic potential of this kind of open-source system, within a perspective of a wireless sensor network constituted of several nodes. Temperature and pH sensors calibration has been performed following the methodology described in the previous paper, and the results are presented in Figure 5a,b for temperature and pH sensors respectively. Both have shown high correlation with the data obtained from the reference (R^2^ = 0.999, *p* < 0.001).

The system utilizes a four-hour cycle for data acquisition and transmission, more than appropriate for wine fermentation dynamics. The nodes perform the reading of the sensors (16 mA peak) for 15 s and data transmission to the coordinator unit (5 mA peak) for 5 s, and then go into sleep mode with minimal power consumption (10 μA) for the remaining cycle time. The nodes were equipped with a full capacity 2300 mA Li-Po battery, but to estimate the available battery life, 80% of capacity was considered (1800 mAh), giving an approximate autonomy of 130 days. This autonomy appears to be optimal in enological applications, and the user can easily remotely control battery level in real-time, ensuring enough time to replace discharged batteries. Figure 5c presents the power supply test results of the WineDuino system compared to the performance of the previous system, showing about 60 extra days of operating time. After this preliminary test phase in the laboratory, the system will be tested in cellar conditions for the 2014 vintage.

Regarding WineDuino system cost analysis, Table 1 presents the single component costs of the node unit and coordinator unit. The prices are VAT included and are taken from an online store [11], specializing in low-cost sensors and open-source hardware. The price is about 135 € for each node (hardware and sensors) and 130 € for the coordinator unit, whereas the WSBN first prototype [5] was much more expensive, approximately double. Moreover the WSBN developed with a commercial partner, was customized to measure only temperature and pH data, whereas the WineDuino microcontroller provides a large number of free channels to potentially manage other types of sensors.

The comparative analysis of the two systems has shown excellent performances by both, however WineDuino proves best for energy consumption and as regards costs. The open-source technology provides easy replication of this system, allowing modification or enhancement by anyone, features that make it an excellent tool for any experimentation or research. However, from a commercial point of view, it is a weak point given that most companies show disinterest in a non-proprietary hardware and software product, because it could be easily copied, becoming a bad investment. This is one of the most limiting factors to the diffusion of open-source technology and leads to a strong presence of proprietary technologies on the market.

## 4. Conclusions/Outlook

In the last years, WSNs have been widely used for different monitoring solutions both in the vineyard [12–15] and cellar [1–5,16]. As reported by Jones and Davis [17], temperature is a critical factor to control during alcoholic fermentation, but different stages of the winemaking process require the monitoring of many other parameters. Moreover, the development and installation of a monitoring system on steel vats would be relatively simple, but the wooden matrix of barrels could cause serious problems, both in terms of positioning and obstacle for barrel management practices, such as sampling, filling or movement. This paper presents an open-source, low-cost WSN applicable to every phase of the wine production process, without any impact on cellar management. The WineDuino system characteristics were evaluated considering the improvements over a previous prototype proposed by the authors [5]. The system is ready to use and easy to install, just replacing the common barrel bung. The power consumption test shows high performance in terms of battery life, ensuring up to 130 days of operating time. WineDuino node is less expensive than the first prototype, and presents no obstacle to cellar management, thanks to its non-invasive size and wireless technology. The system provides high flexibility and customization features, allowing significant opportunity to monitor every critical stage of the wine production chain. WineDuino can thus be a useful DSS (Decision Support System) for the winemaker to follow a Precision Enology approach in quality production. Furthermore, it could easily incorporate a large number of sensors with small changes to the firmware, offering a versatile and expandable tool able to supply a real-time and low-cost wireless monitoring system suitable for many food productions. Future research will include a market study to assess other potential sensors for the system, in order to monitor interesting parameters of all stages of the winemaking production chain. The monitoring needs and critical points of other food processes will also be explored, such as the dairy industry and olive oil production, for the purpose of realizing a customizable monitoring system not exclusively dedicated to wine.

## Figures and Tables

**Table 1.** WineDuino system cost analysis.

**WineDuino Node**			**Coordinator Unit**		
	
**Component**	**Hardware**	**Price (€)**	**Component**	**Hardware**	**Price (€)**
Microcontroller board	Arduino Mini Pro	10.59	Microcontroller board	Raspberry Pi–Model B–512MB	37.82

Radio module	Xbee S1	36.20	Radio module	Xbee S1	36.20

Sensor Shield	Prototype	30.00	USB WiFi dongle	USB WiFi (802.11b/g/n) Module with Antenna	18.91

pH probe	3550–ASP200-2-1M-BNC pH Lab Electrode	29.68	USB 3G dongle	Unlocked 1901F-1 HSDPA USB2.0 GPRS WCDMA 3G Wireless USB Modem Dongle	20.00

Temperature probe	DS18B20	9.98	USB Xbee dongle	XBee–USB Board	20.53

Case	IP65	5.00	Case	IP65	5.00

Power Supply	Li-Po 3.7 V 2300 mAh	7.69	Power Supply	Power supply adapter AC/DC 5V	10.59

Dipstick	stainless steel (AISI 310)	5.00	-	-	-

Total cost (approximately)		135.00	Total cost (approximately)		150.00
